# Dichloroacetate (DCA) and Cancer: An Overview towards Clinical Applications

**DOI:** 10.1155/2019/8201079

**Published:** 2019-11-14

**Authors:** Tiziana Tataranni, Claudia Piccoli

**Affiliations:** ^1^Laboratory of Pre-Clinical and Translational Research, IRCCS-CROB, Referral Cancer Center of Basilicata, Rionero in Vulture (Pz), 85028, Italy; ^2^Department of Clinical and Experimental Medicine, University of Foggia, Foggia 71121, Italy

## Abstract

An extensive body of literature describes anticancer property of dichloroacetate (DCA), but its effective clinical administration in cancer therapy is still limited to clinical trials. The occurrence of side effects such as neurotoxicity as well as the suspicion of DCA carcinogenicity still restricts the clinical use of DCA. However, in the last years, the number of reports supporting DCA employment against cancer increased also because of the great interest in targeting metabolism of tumour cells. Dissecting DCA mechanism of action helped to understand the bases of its selective efficacy against cancer cells. A successful coadministration of DCA with conventional chemotherapy, radiotherapy, other drugs, or natural compounds has been tested in several cancer models. New drug delivery systems and multiaction compounds containing DCA and other drugs seem to ameliorate bioavailability and appear more efficient thanks to a synergistic action of multiple agents. The spread of reports supporting the efficiency of DCA in cancer therapy has prompted additional studies that let to find other potential molecular targets of DCA. Interestingly, DCA could significantly affect cancer stem cell fraction and contribute to cancer eradication. Collectively, these findings provide a strong rationale towards novel clinical translational studies of DCA in cancer therapy.

## 1. Introduction

**Figure 1:**
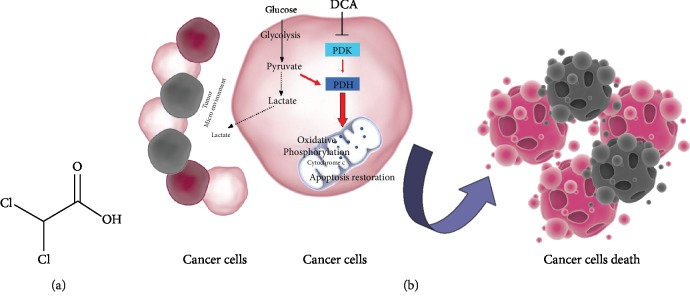
(a) Chemical structure of DCA. (b) Mechanism of action of DCA: PDK: pyruvate dehydrogenase kinase; PDH: pyruvate dehydrogenase. Black dotted lines, biochemical processes inhibited by DCA; Red arrows, metabolic pathways activated by DCA.

Cancer is one of the leading causes of death worldwide. Despite the significant progression in diagnostic and therapeutic approaches, its eradication still represents a challenge. Too many factors are responsible for therapy failure or relapse, so there is an urgent need to find new approaches to treat it. Apart from the typical well-known properties featuring malignant cells, including abnormal proliferation, deregulation of apoptosis, and cell cycle [1, 2], cancer cells also display a peculiar metabolic machine that offers a further promising approach for cancer therapy [3–5]. Our group had already suggested the importance of a metabolic characterization of cancer cells to predict the efficacy of a metabolic treatment [6]. Drugs able to affect cancer metabolism are already under consideration, showing encouraging results in terms of efficacy and tolerability [7]. In the last decade, the small molecule DCA, already used to treat acute and chronic lactic acidosis, inborn errors of mitochondrial metabolism, and diabetes [8], has been largely purposed as an anticancer drug. DCA is a 150 Da water-soluble acid molecule, analog of acetic acid in which two of the three hydrogen atoms of the methyl group have been replaced by chlorine atoms (Figure 1(a)) [9]. DCA administration in doses ranging from 50 to 200 mg/Kg/die is associated to a decrease of tumour mass volume, proliferation rate, and metastasis dissemination in several preclinical models [10]. Our group had already observed an inverse correlation between DCA ability to kill cancer cells and their mitochondrial respiratory capacity in oral cell carcinomas [11]. Moreover, we recently described DCA ability to affect mitochondrial function and retarding cancer progression in a pancreatic cancer model [12]. To date, consistent data from clinical trials and case reports describing DCA administration in cancer patients are available [13–16], but, despite the growing body of literature sustaining the efficacy of DCA against cancer, it is not under clinical use yet. This review is aimed at summarizing the very recent reports suggesting the employment of DCA in cancer therapy, in combination with chemotherapy agents, radiotherapy, and other chemical or natural compounds showing anticancer properties. Moreover, we described data about new purposed pharmacological formulations of DCA able to avoid side effects and ameliorate drug bioavailability and efficacy, further encouraging its possible clinical employment. Finally, we reviewed latest findings suggesting other potential mechanisms of action of DCA, including new data about its aptitude to affect cancer stem cell fraction.

## 2. DCA and Cancer: Mechanism of Action

The potential efficacy of DCA in cancer therapy comes from metabolic properties of cancer cells, typically characterized by increased glycolytic activity and reduced mitochondrial oxidation, regardless of oxygen availability, the well-known Warburg effect [17]. The excessive glycolysis and the resulting lactate overproduction provoke a state of metabolic acidosis in tumour microenvironment [18]. Glycolysis-derived lactate is taken up by surrounding cells to support tumour growth and inhibits apoptotic cell death mechanisms [19, 20]. Several enzymes involved in glycolysis regulate apoptosis, and their overexpression in cancer cells contributes to apoptosis suppression [21]. In this setting, salts of DCA selectively target cancer cells shifting their metabolism from glycolysis to oxidative phosphorylation by inhibition of pyruvate dehydrogenase kinase (PDK), the inhibitor of pyruvate dehydrogenase (PDH) [10]. PDH activation fosters mitochondrial oxidation of pyruvate and disrupts the metabolic advantage of cancer cells. Mitochondrial DNA mutations, often occurring in tumorigenesis and resulting in respiratory chain dysfunction [22, 23], make malignant cells unable to sustain cellular energy demand. Furthermore, reducing lactate production, DCA counteracts the acidosis state of tumour microenvironment, contributing to the inhibition of tumour growth and dissemination [24]. The delivery of pyruvate into mitochondria causes organelles remodelling resulting in an increased efflux of cytochrome c and other apoptotic-inducing factors and upregulation of ROS levels with a consequent reduction of cancer cell viability [9] (Figure 1(b)).

## 3. Side Effects and Limitations to DCA Employment

Clinical employment of DCA is available in both oral and parenteral formulations, and doses range from 10 to 50 mg/Kg/die [25]. No evidence of severe hematologic, hepatic, renal, or cardiac toxicity confirms DCA safety [26]. Common gastrointestinal side effects often occur in a percentage of patients treated with DCA [15]. The best-known limitation to DCA administration, observed both in preclinical and in clinical studies, is peripheral neuropathy [27]. The selectivity of DCA-induced damage for the nervous system may be due to the lack of well-equipped machinery able to handle a more sustained oxidative phosphorylation in cells producing ATP mostly via glycolysis [28]. The resulting mitochondrial overload compromises the antioxidant systems' efficiency, unable to face the excessive amount of ROS. In this setting, the contemporary administration of antioxidants should represent a further strategy to minimize DCA-induced neuropathy [27]. The expression and the activity of glutathione transferase zeta1 (GSTZ1), the first enzyme responsible for DCA clearance, may influence the entity of damage. Nonsynonymous functional single-nucleotide polymorphisms (SNPs) in human GSTZ1 gene give rise to different haplotypes that are responsible for a different DCA kinetic and dynamics. A clear association between GSTZ1 haplotype and DCA clearance has been demonstrated. On this basis, a personalized DCA dosage, not only based on body weight, may minimize or prevent adverse effects in patients chronically treated with this drug [29]. The occurrence of neuropathy is associated to DCA chronic oral administration and is a reversible effect, limited to the time of treatment [30]. The intravenous route reduces, therefore, the potential for neurotoxicity and let the achievement of higher drug concentrations bypass the digestive system [13].

Since DCA is among water disinfection by-products found in low concentrations in drinking water, its potential carcinogenicity is under evaluation. Studies performed in mouse models associate DCA early-life exposure to an increased incidence of hepatocellular tumours [31]. It is conceivable that persistent changes in cell metabolism induced by DCA may produce epigenetic effects. Long-term induction of PDH and other oxidative pathways related to glucose metabolism could contribute to increase reactive oxygen species and mitochondrial stress [27]. However, no evidence of carcinogenetic effect is reported in clinical studies, when DCA is administered in cancer therapy.

## 4. Synergistic Effect of DCA and Chemotherapeutic Agents

Combining different drugs is a well-accepted strategy to produce a synergistic beneficial effect in cancer therapy, reducing drug dosage, minimizing toxicity risks, and overcoming drug resistance. Coadministration of DCA and traditional chemotherapeutic agents has been purposed and tested in several cancer models (Table 1). DCA treatment seems to improve the efficacy of chemotherapy by inducing biochemical and metabolic alterations, resulting in significant changes of cancer cells' energetic balance. A study performed in non-small-cell lung cancer (NSCLC) showed both *in vitro* and *in vivo* that coadministration of DCA with paclitaxel increased the efficiency of cell death through autophagy inhibition [32]. An effective combination of DCA and doxorubicin (DOX) was tested in HepG2 cells, demonstrating the ability of DCA to disrupt cellular antioxidant defences, thus favouring oxidative damage in turn triggered by DOX treatment [33]. There is a strong association between PDK overexpression and chemoresistance; thus, it is conceivable that PDK inhibition might help to resensitize cancer cells to drugs. PDK2 isoform overexpression was associated to paclitaxel resistance in NSCLC. Interestingly, DCA combination with paclitaxel was more effective in killing resistant cells than either paclitaxel or DCA treatment alone [34]. Similarly to NSCLC, an interesting *in vivo* study performed in advanced bladder cancer showed an increased expression of PDK4 isoform in high grade compared to lower-grade cancers and cotreatment of DCA and cisplatin dramatically reduced tumour volumes as compared to either DCA or cisplatin alone [35]. A recent study confirmed the ability of DCA to revert PDK4-related chemoresistance also in human hepatocellular carcinoma (HCC) [36].

## 5. Synergistic Effect of DCA and Other Potential Anticancer Drugs

A consistent body of literature suggests positive effects of DCA coadministration with compounds currently employed to treat other diseases but showing anticancer properties in several cancer models (Table 2). Contemporary administration of DCA and the antibiotic salinomycin, recently rediscovered for its cytotoxic properties as a potential anticancer drug, has been tested in colorectal cancer cell lines. Their treatment seems to exert a synergistic cytotoxic effect by inhibiting the expression of proteins related to multidrug resistance [37]. Cancer cells lacking metabolic enzymes involved in arginine metabolism may result to sensitivity to arginase treatment. Interestingly, a combined administration of recombinant arginase and DCA produces antiproliferative effects in triple-negative breast cancer, due to the activation of p53 and the induction of cell cycle arrest [38]. COX2 inhibitors, primarily used as anti-inflammatory drugs, have been recently suggested as antitumor drugs because of their antiproliferative activity. An intriguing study performed in cervical cancer cells showed the inability of DCA to kill cervical cancer cells overexpressing COX2 and demonstrated that COX2 inhibition by celecoxib makes cervical cancer cells more sensitive to DCA both *in vitro* and *in vivo* experiments [39]. Since DCA fosters oxidative phosphorylation by decreasing glycolytic activity, the combination of DCA with other drugs enhancing a state of glucose dependence may be a promising strategy. Such an approach has been tested in head and neck cancer in which the administration of propranolol, a nonselective beta-blocker able to affect tumour cells' mitochondrial metabolism, produced glycolytic dependence and energetic stress, making cells more vulnerable to DCA treatment [40]. Similar results were obtained in melanoma cells in which the administration of retinoic acid receptor *β* (RAR*β*) inhibitors confer sensitization to DCA [41]. A positive effect of DCA coadministration with metformin, a hypoglycaemic drug widely used to treat diabetes was demonstrated in a preclinical model of glioma [42] as well as in a low metastatic variant of Lewis lung carcinoma (LLC) [43]. Jiang and colleagues investigated the effects of phenformin, a metformin analog, and DCA in glioblastoma, demonstrating that contemporary inhibition of complex I and PDK by phenformin and DCA, respectively, decreased self-renewal and viability of glioma stem cells (GSCs), thus suggesting their possible employment to affect cancer stem cell fraction [44].

## 6. Combined Use of DCA and Natural Compounds

The clinical employment of natural compounds represents a promising novel approach to treat several diseases [45]. An increasing body of literature supports the detection, among natural compounds, of biologically active substances isolated by plants, mushrooms, and bacteria or marine organism that show beneficial effects for human health [46–48]. The assumption of natural compounds or their derivatives seems to represent an encouraging approach to prevent cancer initiation or recurrence, and it is generally called chemoprevention [49]. Moreover, natural substances produce beneficial effects in cancer therapy when coadministered with other drugs, showing their ability to overcome drug resistance, to increase anticancer potential, and to reduce drug doses and toxicity [50, 51]. Interestingly, the coadministration of DCA and natural compounds has been recently purposed. A study investigated the combined effect of DCA with essential oil-blended curcumin, a compound with beneficial properties both in prevention and treatment of cancer [52], demonstrating an anticancer potential against HCC [53]. In particular, the combination of both compounds synergistically reduced cell survival, promoting cell apoptosis and inducing intracellular ROS generation. Betulin, a natural compound isolated from birch bark, is already known for its antiproliferative and cytotoxic effects against several cancer cell lines [54–56]. An *in vitro* investigation of the antitumor activity of betulin derivatives in NSCLC confirmed its ability to inhibit *in vivo* and *in vitro* growth of lung cancer cells, blocking G2/M phase of the cell cycle and inducing caspase activation and DNA fragmentation. Interestingly, betulin derivative Bi-L-RhamBet was able to perturb mitochondrial electron transport chain (ETC), inducing ROS production. Given the property of DCA to increase the total oxidation of glucose in mitochondria via the Krebs cycle and ETC, the authors combined Bi-L-RhamBet with DCA, demonstrating its significant potentiated cytotoxicity [57].

## 7. DCA and Radiosensitization

Radiotherapy represents a further strategy to treat cancer and provides a local approach by the administration of high-energy rays [58]. The main effect of radiation is the induction of ROS with a consequent DNA damage, chromosomal instability, and cell death by apoptosis [59]. However, several tumours show or develop radioresistance that is responsible for radiotherapy failure and high risk of tumour recurrence or metastasis [60]. Several factors may be responsible of radioresistance [61]. Among these, hypoxia, a common condition of tumour microenvironment characterized by low oxygen levels and reduced ROS species generation, can block the efficacy of ionizing radiations [62]. Increasing tumour oxygenation so to favour a considerable amount of ROS [63] or directly induce ROS production may therefore represent a strategy to increase radiosensitization [64, 65]. In this setting, DCA administration, known to induce ROS production [11, 66], could represent a strategy to overcome tumour radioresistance. Moreover, metabolic alterations featuring cancer development are known to affect radiosensitivity [67, 68]. Therefore, targeting cancer metabolic intermediates may represent a strategy to improve a selective cancer response to irradiation [69]. The efficacy of DCA to increase radiation sensitivity has been already demonstrated both in glioblastoma cells [70] and in oesophageal squamous cell carcinoma [71]. More recently, it was demonstrated that DCA increases radiosensitivity in a cellular model of medulloblastoma, a fatal brain tumour in children, inducing alterations of ROS metabolism and mitochondrial function and suppressing DNA repair capacity [72]. Since the role of immunotherapy in the restoration of the immune defences against tumour progression and metastasis is arousing great attention in the last years [73], Gupta and Dwarakanath provided a state of the art of the possible effects of glycolytic inhibitors, including DCA, on tumour radiosensitization, focusing their attention on the interplay between metabolic modifiers and immune modulation in the radiosensitization processes [74]. Interestingly, they reported the ability of DCA to promote immune stimulation through the inhibition of lactate accumulation, further sustaining its utilization as adjuvant of radiotherapy.

## 8. DCA and New Drug Formulations

**Figure 2:**
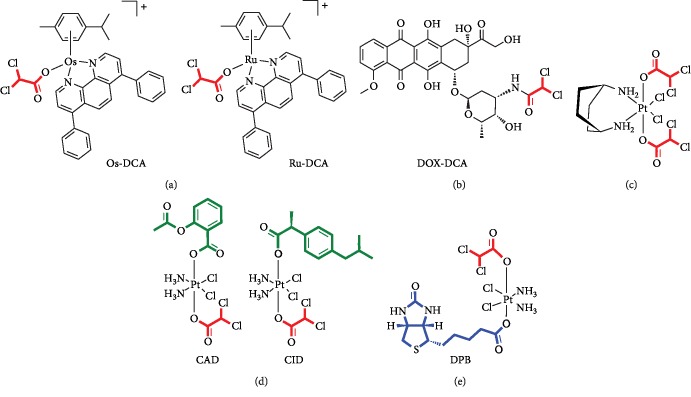
New drug formulations containing DCA. (a) Schematic representation of Os-DCA and Ru-DCA complexes [81]. (b) Doxorubicin (DOX)-DCA complex [83]. (c) Dual action Pt prodrugs of kiteplatin and DCA [84]. (d) Examples of triple action Pt(_IV_) derivatives of cisplatin containing DCA (red), derivatives of cisplatin (black), and COX inhibitors (green) [85]. (e) Chemical structure of DPB containing DCA (red), biotin (blue), and Platinum (Pt) complex (black) [86].

There is a growing interest in designing new drug formulations so to improve drug delivery, increasing the efficacy and reducing the doses and consequently undesirable effects. In this setting, drug delivery systems (DDSs) represent a new frontier in the modern medicine [75]. DDSs offer the possibility to create a hybrid of metal-organic frameworks (MOFs), combining the biocompatibility of organic system to the high loadings of inorganic fraction [76]. Several lines of evidence suggest an efficient functionalization of nanoparticles with DCA. Lazaro and colleagues [77] explored different protocols for DCA functionalization of the zirconium (Zr) terephthalate (UiO-66) nanoparticles. They demonstrated the cytotoxicity and selectivity of the same DDSs against different cancer cell lines. Moreover, they excluded a possible response of the immune system to DCA-MOF *in vitro*. The same group later showed the possibility to load Zr MOFs with a second anticancer drug, such as 5-fluorouracil (5-FU), so to reproduce the synergistic effect of the two drugs [78]. Zirconium-based MOF loaded with DCA was also purposed as an attractive alternative to UiO-66, showing selective *in vitro* cytotoxicity towards several cancer cell lines and a good toleration by the immune system of several species [79]. Recently, Štarha et al. [80] synthesized and characterized, for the first time, half-sandwich complexes containing ruthenium or osmium and DCA (Figure 2(a)). Both Ru-dca and Os-DCA complexes were screened in ovarian carcinoma cell lines, demonstrating to be more cytotoxic than cisplatin alone. Both complexes were able to induce cytochrome c (Cytc) release from mitochondria, an indirect index of apoptosome activation and seemed to be less toxic towards healthy primary human hepatocytes, thus indicating selectivity for cancer over noncancerous cells. Promising results were also obtained in triple-negative breast cancer cells [81]. Rhenium (I)-DCA conjugate has demonstrated an efficient penetration into cancer cells and a selective accumulation into mitochondria, inducing mitochondrial dysfunction and metabolic disorders [82]. In the recent years, several multiactive drugs have been designed to contemporary target different intracellular pathways using a single formulation. A safe, simple, reproducible nanoformulation of the complex doxorubicin-DCA (Figure 2(b)) was successfully tested in a murine melanoma model, showing an increase in drug-loading capability, lower side effects, and enhanced therapeutic effect [83]. Dual-acting antitumor Pt (IV) prodrugs of kiteplatin with DCA axial ligands have been synthesized (Figure 2(c)), characterized, and tested in different tumour cell lines and *in vivo* [84]. To overcome cancer resistance, triple action Pt (IV) derivatives of cisplatin have been proposed as new potent anticancer agents, able to conjugate the action of cisplatin, cyclooxygenase inhibitors, and DCA (Figure 2(d)) [85]. A novel complex containing DCA, Platinum, and Biotin (DPB) has been successfully tested, exhibiting multifacet antitumor properties (Figure 2(e)). Authors demonstrated the ability of such a prodrug to affect energy metabolism, to promote apoptosis, and to interact with DNA. The high selectivity of biotin for cancer cells minimizes the detrimental effects on normal cells and improves the curative effect on tumours [86]. Features and experimental evidence of the main classes of compounds are summarized in Table 3.

## 9. Other Proposed Mechanisms of Action of DCA

**Figure 3:**
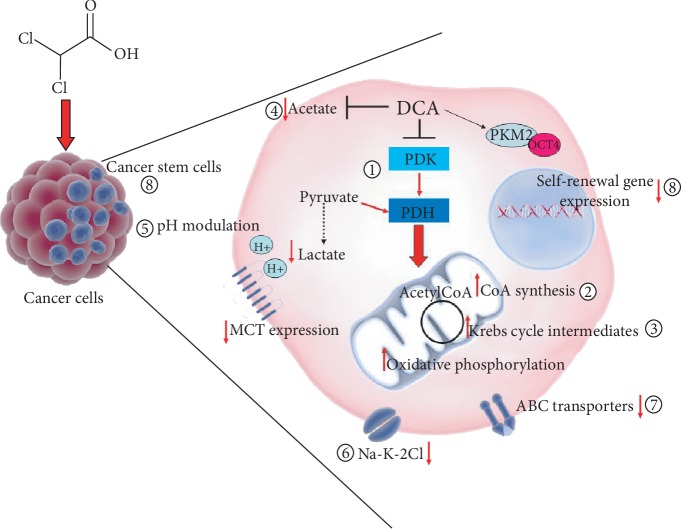
Other proposed mechanisms of action of DCA. DCA's main mechanism is to inhibit pyruvate dehydrogenase kinase (PDK), leading to pyruvate dehydrogenase (PDH) activation and fostering oxidative phosphorylation (1). DCA also increases each Krebs cycle intermediate concentration (2) [87]. DCA induces cell toxicity via de novo synthesis of CoA (3) [88]. DCA may antagonize acetate (4) [90]. DCA modulates intracellular acidification (5) [93, 94]. DCA inhibits Na-K-2Cl cotransporter (6) [96]. DCA downregulates gene and protein expression of ABC transporters (7) [97]. DCA reduces the expression of self-renewal-related genes and affects cancer stem cell fraction (8) [99].

The metabolic shift from glycolysis to glucose oxidation due to the inhibition of PDK and the consequent activation of PDH is the best-known and well-accepted molecular effect of DCA administration. The consequent biochemical alterations, including ROS increase and mitochondrial membrane potential variation, may be responsible for proliferation arrest and cancer cell death, thus explaining DCA beneficial potential in cancer treatment [9]. However, the molecular intermediates activated after DCA administration are still unknown. It is conceivable that such a small molecule might directly or indirectly affect other cellular and molecular targets (Figure 3), displaying other mechanisms of action, so to explain its efficacy also in cellular models where it does not produce the expected metabolic shift [12]. A proteomic approach applied to cells of lung cancer demonstrated the ability of DCA to increase the concentration of every TCA intermediate while it did not affect glucose uptake or the glycolytic process from glucose to pyruvate [87]. In the attempt to shed light to DCA mode of action, Dubuis and colleagues used a metabolomics-based approach on several ovarian cancer cell lines treated with DCA and found a common marked depletion of intracellular pantothenate, a CoA precursor, as well as a concomitant increase of CoA, thus suggesting DCA ability to increase CoA de novo biosynthesis. Since high concentrations of CoA resulted to be toxic for cells, this metabolic effect could be responsible of cancer cell toxicity mediated by DCA [88]. A very recent work by El Sayed et al. introduced a novel evidence-based hypothesis, suggesting that DCA efficiency against cancer may derive from its ability to antagonize acetate [89], known to be an energetic substrate for glioblastoma and brain metastases, able to enhance DNA, RNA, and protein synthesis and posttranslational modifications, thus favouring cell proliferation and cancer progression. Moreover, high acetate levels are associated to anticancer drug resistance [90]. It has been shown that DCA is able to revert metabolic alterations induced by acetate by restoring physiological serum levels of lactate and free fatty acid and potassium and phosphorus concentration. According to the authors, thanks to a structural similarity to acetate, DCA could inhibit metabolic effects driven by acetate, responsible for cancer cell growth and chemoresistance [89]. Another possible additional effect of DCA could be pH modulation. pH level modulation is known to affect proliferation and apoptosis processes [91] as well as chemotherapy sensitivity [92]. DCA treatment may both increase and reduce intracellular pH. A secondary effect of pyruvate redirecting into the mitochondria by DCA would be lactate reduction and a consequent increase in intracellular pH. On the other side, DCA is able to decrease the expression of monocarboxilate transporters and V-ATPase with a consequent reduction of pH, and this especially occurs in tumour cells, expressing higher amount of these carriers, compared to normal counterparts [93]. Given the ability to induce rapid tumour intracellular acidification, Albatany et al. [94] speculated about a possible employment of DCA as a tracker in *in vivo* imaging of a glioblastoma murine model and supported a therapeutic use of DCA since intracellular acidification is known to induce caspase activation and DNA fragmentation of cancer cells [95]. Animal models allow to identify a possible further molecular target of DCA. Experiments performed in rats highlighted the ability of DCA to inhibit the expression of the renal cotransporter Na-K-2Cl (NKCC) in the kidney of rats [96]. As NKCC is an important biomarker of extracellular and intracellular ion homeostasis regulation and participates in cell cycle progression, it plays an important role in cancer cell proliferation, apoptosis, and invasion. Belkahla et al. [97] investigated the interplay between metabolism targeting and the expression of ABC transporters, responsible for drug export from cells and a consequent multidrug resistance, and found that DCA treatment is able to reduce gene and protein expression of ABC transporters in several tumour cells expressing wild type p53, both *in vitro* and *in vivo* [98]. It has been already demonstrated the ability of DCA to induce differentiation through the modulation of PKM2/Oct4 interaction in glioma cells [99]. The resulting reduction of Oct4 transcription levels was associated to a reduction of stemness phenotype and a significant increased sensitivity to cell stress. This observation lets to hypothesize a potential role of DCA against cancer stem cells (CSCs).

## 10. DCA and Cancer Stem Cells

There is a growing interest in targeting cancer stem cells (CSCs) which seem to be the main responsible for tumour relapse [100]. CSCs share the ability of self-renewal with normal stem cells and can give rise to differentiating cells, responsible for tumour initiation as well as malignant progression [101]. A low proliferation rate and specific metabolic profile contribute to make CSCs resistant to conventional chemotherapy [102]. An urgent need emerged in the developing of new therapeutic agents able to affect cancer stem cell viability [103] in order to completely eradicate the tumour mass. An extensive body of literature is focusing the attention on the metabolic phenotype of CSCs, which seem to differ from differentiated cancer cells and could represent a therapeutic target [104–108]. In this setting, the possible sensitivity of CSC fraction to DCA has been hypothesized and tested in different cancer models. Embryonal carcinoma stem cells represent one of the more appropriate models for the study of CSC maintenance and differentiation and the identification of drugs and molecules able to modulate these processes [109]. Studies performed on embryonic stem cells (ESCs) constitute preliminary important proofs supporting a possible efficacy of DCA [110]. Interestingly, DCA treatment of ESCs promotes loss of pluripotency and shifts towards a more active oxidative metabolism, accompanied by a significant decrease in HIF1a and p53 expression [111]. Vega-Naredo et al. [112] described the importance of mitochondrial metabolism in directing stemness and differentiation in such a model. They characterized the metabolic profile of stem cell fraction and guessed the less susceptibility of stem phenotype to mitochondrial-directed therapies. Forcing CSCs towards an oxidative metabolism by DCA treatment enabled departure from stemness to differentiation. Several reports support the existence of CSCs in glioma [113, 114], and the efficiency of DCA to hit CSCs has been extensively evaluated in such a cancer type, so difficult to treat with conventional therapies and characterized by low rates of survival. Already in 2010, Michelakis and colleagues had suggested, both *in vitro* and *in vivo*, DCA ability to induce apoptosis of cancer stem cell fraction [26]. A rat model of glioma, recapitulating several features of human glioblastoma, confirmed the efficacy of DCA to potentiate apoptosis of glioma CSCs, characterized by a significant glycolytic pathway overstimulation, compared to normal stem cells [115]. Also, Jiang et al. investigated the effect of DCA on the small population of glioma stem cells (GSCs) isolated from glioblastoma, demonstrating a reduction of self-renewal properties and an increase in cell death percentage [44]. Moreover, an *in vivo* test on mice bearing DCA-treated GSC-derived xenografts showed a significant increase in overall survival. DCA treatment was also tested in melanoma stem cell fraction, and the derived bioenergetics modulation was able to counteract protumorigenic action of a c-Met inhibitor [116]. A very recent work performed on human hepatocellular carcinoma identified PDK4 overexpression in spheres originated from cancer cells, featuring a defined stem-like phenotype. Interestingly, DCA treatment was able to reduce cell viability both of cancer-differentiated cells and cancer stem cells and reversed chemoresistance to conventional therapy [36]. Our group has recently experienced the ability of DCA to reduce the expression of cancer stem cell markers CD24/CD44/EPCAM in a pancreatic cancer cell line as well as to compromise spheroid formation and viability [12], further corroborating data obtained in other cancer models. Together with chemoresistance, also radioresistance represents a limit to an efficient cancer treatment, and CSCs seem to be responsible for such refractoriness [117]. Sun et al. demonstrated the ability of DCA to increase radiosensitivity of medulloblastoma cells by affecting stem-like clones, reducing the expression percentage of CD133-positive cells and reducing sphere formation [72]. Moreover, in the same cellular model, they showed an altered mechanism of DNA repair induced by DCA able to explain the increased effectiveness of radiotherapy.

## 11. Conclusions

Targeting cancer cell metabolism represents a new pharmacological approach to treat cancer. DCA ability to shift metabolism from glycolysis to oxidative phosphorylation has increased the interest towards this drug already known for its anticancer properties. The evidence accumulated in the last years confirms the capability of DCA to overcome chemo, radioresistance in several cancer types and lets to hypothesize additional cellular targets able to explain its skill to kill cancer cells. There is a need to design further clinical studies now limited to poor-prognosis patients with advanced, recurrent neoplasms, already refractory to other conventional therapies. Its potential efficacy against cancer stem cells as well as the development of new drug formulations takes us closer to reach an effective clinical employment of DCA.

## Figures and Tables

**Table 1:** List of reports suggesting beneficial effect of DCA and chemotherapy coadministration in several types of cancers.

Tumour entity	Model system	Chemotherapy drug coadministered with DCA	Mechanism of action	Outcome	References
Lung cancer	A549-H1975 cell lines/xenograft model	Paclitaxel	Autophagy inhibition	Efficacious cancer chemotherapy sensitization	[32]
Hepatocarcinoma	HepG2 cell line	Doxorubicin	Antioxidant defence disruption	Increased cellular damage by oxidative stress induction	[33]
Lung cancer	A549 cell line	Paclitaxel	Increased chemosensitivity through PDK2 inhibition	Paclitaxel resistance overcome	[34]
Bladder cancer	HTB-9, HT-1376, HTB-5, HTB-4 cell lines/xenograft model	Cisplatin	Increased chemosensitivity through PDK4 inhibition	Increased cell death of cancer cells and potential therapeutic advantage	[35]
Hepatocarcinoma	Sphere cultures from HepaRG and BC2 cell lines	Cisplatin, sorafenib	Increased chemosensitivity through PDK4 inhibition	Improved therapeutic effect of chemotherapy by mitochondrial activity restoration	[36]

**Table 2:** List of drugs with their main function tested in combination with DCA in several cancer models.

Drug	Main function	Tumour entity	Model system	Outcome	References
Salinomycin	Antibiotic	Colorectal cancer	DLD-1 and HCT116 cell lines	Inhibition of multidrug resistance-related proteins	[37]
Arginase	Arginine metabolism	Breast cancer	MDA-MB231 and MCF-7/xenograft model	Antiproliferative effect due to p53 activation and cell cycle arrest	[38]
COX2 inhibitors	Inflammation	Cervical cancer	HeLa and SiHa cell lines/xenograft model	Cancer cell growth suppression	[39]
Propranolol	Beta-blocker	Head and neck cancer	mEERL and MLM3 cell lines/C57Bl/6 mice	Glucose dependence promotion and enhancement of chemoradiation effects	[40]
RAR*β* inhibitors	Vitamin A metabolism	Melanoma	ED-007, ED-027, ED-117, and ED196 cell lines	Glucose dependence promotion and sensitization to DCA	[41]
Metformin	Diabetes	Glioma, Lewis lung carcinoma	Xenograft model; LLC/R9 cells	Prolonged lifespan of mice with glioma; severe glucose dependency in tumour microenvironment	[42, 43]
Phenformin	Diabetes	Glioblastoma	Glioma stem cells/xenograft model	Self-renewal inhibition of cancer stem cells	[44]

**Table 3:** Properties of the main classes of DCA drug formulations tested in cancer cell lines and *in vivo* models with experimental evidence related.

Class of drug formulation	Features	*In vitro* tests	*In vivo* tests	Experimental evidence	References
Metal-DCA frameworks (no platinum)	Metal ions linked to organic ligands into porous scaffolds	MCF-7/MDA-MB-231 (breast) HeLa/LO2 (cervix) A2780 (ovary) A549/NCl-H1229 (lung)	Breast mouse models	Biocompatibility selective cytotoxicity Immune system compatibility Low mutagenicity	[77–82]

Doxorubicin-DCA conjugate	Complexes of DCA and chemotherapy drugs	B16F10 (melanoma)	Sarcoma and melanoma mouse models	Selective cytotoxicity safety In vivo antitumour efficiency	[83]

Platinum prodrugs with DCA	Platinum core associated to DCA and others drugs	MCF-7 (breast) LoVo/HCT-15/HCT116 (colon) A549 (lung) BxPC3/PSN-1 (pancreas) A375 (melanoma) BCPAP (thyroid) HeLa (cervix) HepG2 (hepatocarcinoma)	Lung carcinoma mouse models	Selective cytotoxicity multiple action Increased cellular uptake	[84–86]
